# Poly[aqua­(μ_1,1_-azido)(μ-3*H*-1,2,3-tri­azolo[4,5-*b*]pyridin-3-olato)cobalt(II)]

**DOI:** 10.1107/S1600536810023809

**Published:** 2010-06-26

**Authors:** Jiong-Peng Zhao, Fu-Chen Liu

**Affiliations:** a School of Chemistry and Chemical Engineering, Tianjin University of Technology, Tianjin 300191, People’s Republic of China

## Abstract

In the title compound, [Co(C_5_H_3_N_4_O)(N_3_)(H_2_O)]_*n*_, the cobalt ion is coordinated by three N atoms of two organic ligands, two N atoms of two azide anions and one water mol­ecule in a distorted octa­hedral geometry. The metal atoms are connected *via* the ligands into layers, which are further connected by O—H⋯N and O—H⋯O hydrogen bonding.

## Related literature

For the coordination modes of azide anions, see: Zeng *et al.* (2009 ▶). For the preparation and chacterization of metal–azide complexes with different co-ligands, see: Wang *et al.* (2008 ▶).
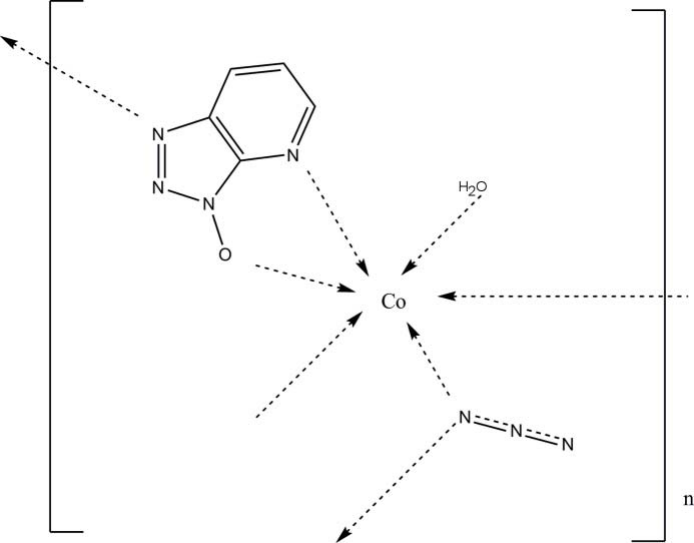


## Experimental

### 

#### Crystal data

[Co(C_5_H_3_N_4_O)(N_3_)(H_2_O)]
*M* *_r_* = 254.09
Monoclinic, 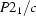

*a* = 7.0891 (14) Å
*b* = 10.122 (2) Å
*c* = 12.685 (4) Å
β = 113.08 (2)°
*V* = 837.4 (4) Å^3^
*Z* = 4
Mo *K*α radiation
μ = 2.04 mm^−1^
*T* = 293 K
0.20 × 0.18 × 0.18 mm

#### Data collection

Rigaku SCXmini diffractometer
Absorption correction: multi-scan (*ABSCOR*; Higashi, 1995 ▶) *T*_min_ = 0.462, *T*_max_ = 1
6902 measured reflections
1469 independent reflections
1352 reflections with *I* > 2σ(*I*)
*R*_int_ = 0.040

#### Refinement

*R*[*F* ^2^ > 2σ(*F* ^2^)] = 0.044
*wR*(*F* ^2^) = 0.112
*S* = 1.09
1469 reflections
144 parameters
H atoms treated by a mixture of independent and constrained refinement
Δρ_max_ = 1.50 e Å^−3^
Δρ_min_ = −0.43 e Å^−3^

### 

Data collection: *SCXmini Benchtop Crystallography System Software* (Rigaku, 2006 ▶); cell refinement: *PROCESS-AUTO* (Rigaku, 1998 ▶); data reduction: *PROCESS-AUTO*; program(s) used to solve structure: *SHELXS97* (Sheldrick, 2008 ▶); program(s) used to refine structure: *SHELXL97* (Sheldrick, 2008 ▶); molecular graphics: *ORTEPIII* (Burnett & Johnson, 1996 ▶) and *PLATON* (Spek, 2009 ▶); software used to prepare material for publication: *SHELXTL* (Sheldrick, 2008 ▶).

## Supplementary Material

Crystal structure: contains datablocks global, I. DOI: 10.1107/S1600536810023809/nc2187sup1.cif

Structure factors: contains datablocks I. DOI: 10.1107/S1600536810023809/nc2187Isup2.hkl

Additional supplementary materials:  crystallographic information; 3D view; checkCIF report

## Figures and Tables

**Table 1:** Hydrogen-bond geometry (Å, °)

*D*—H⋯*A*	*D*—H	H⋯*A*	*D*⋯*A*	*D*—H⋯*A*
O1*W*—H1*WB*⋯N7^i^	0.74 (7)	2.15 (7)	2.894 (6)	178 (7)
O1*W*—H1*WA*⋯O1^ii^	0.84 (8)	1.87 (8)	2.661 (5)	156 (8)

Symmetry codes: (i) 
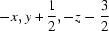
; (ii) 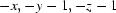.

**Fig. 1.**
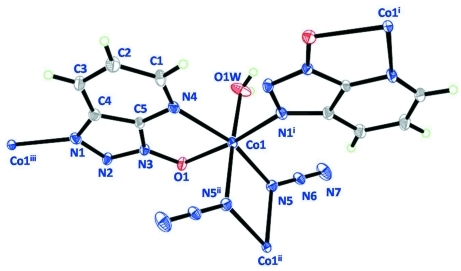
The structure of the complex with labelling and displacement ellipsoids drawn at the 30% probability level. Symmetry codes: i = x,-y-1/2,z-1/2, ii = -x+1,-y-1,-z-1 and iii = x,-y-1/2,z+1/2

**Fig. 2.**
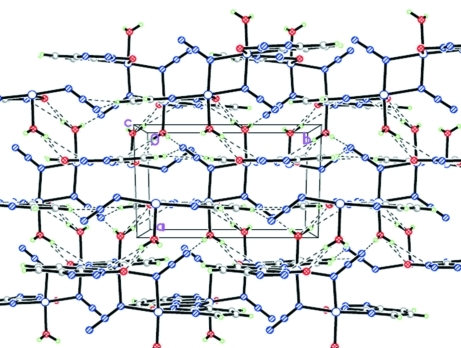
Crystal structure of the title compound with view along the c axis.

## supplementary crystallographic information

### Comment

Azide anion has drawn much attentions because they can coordinate to metal ions in diverse coordination modes (Zeng,*et al.*, 2009). Therefore, several metal azide complexes with different co-ligand has been prepared and characterized (Wang,*et al.*, 2008). As a part on a project of new metal azide coordination polymers the structure of the title compound was determined.

In the crystal structure of the title compound the Co ions are coordinated by two N atoms of two symmetry related azide anion, three N atoms of two symmetry related organic ligands and one water molecule within slightly distorted octahedra (Fig. 1). The Co ions are connected via two end-on bridging thiocyanato anions into chains, that are further be connected into layers by the organic ligands. These layers are located in the b-c-plane and are linked via N-H···O and N-H···N hydrogen bonding to adjacent water molecules and azide anions (Fig. 2).

### Experimental

A mixture of Co(II)nitrate (1.5mmol), 3H-[1,2,3]triazolo[4,5-b]pyridin-3-ol(0.75 mmol), and sodium azide (2mmol), in 10 ml MeOH solvent was sealed in a Teflon-lined stainless-steel Parr bomb that was heated at 413 K for 48 h. Red crystals of the title complex were collected after the bomb was allowed to cool to room temperature. Yield 20% based on metal salt.

### Refinement

Hydrogen atoms of water molecule were added by difference Fourier maps and refined directly. Other hydrogen atoms were included in calculated positions and treated as riding on their parent C atoms with C—H = 0.93Å and *U*_iso_(H) = 1.2*U*_eq_(C).

### Crystal data

**Table:**

[Co(C_5_H_3_N_4_O)(N_3_)(H_2_O)]	*F*(000) = 508
*M**_r_* = 254.09	*D*_x_ = 2.015 Mg m^−^^3^
Monoclinic, *P*2_1_/*c*	Mo *K*α radiation, λ = 0.71073 Å
*a* = 7.0891 (14) Å	Cell parameters from 7903 reflections
*b* = 10.122 (2) Å	θ = 3.1–27.7°
*c* = 12.685 (4) Å	µ = 2.04 mm^−^^1^
β = 113.08 (2)°	*T* = 293 K
*V* = 837.4 (4) Å^3^	Block, red
*Z* = 4	0.2 × 0.18 × 0.18 mm

### Data collection

**Table:**

Rigaku SCXmini diffractometer	1469 independent reflections
Radiation source: fine-focus sealed tube	1352 reflections with *I* > 2σ(*I*)
graphite	*R*_int_ = 0.040
ω scans	θ_max_ = 25.0°, θ_min_ = 3.1°
Absorption correction: multi-scan (*ABSCOR*; Higashi, 1995)	*h* = −8→8
*T*_min_ = 0.462, *T*_max_ = 1	*k* = −12→12
6902 measured reflections	*l* = −15→15

### Refinement

**Table:**

Refinement on *F*^2^	Primary atom site location: structure-invariant direct methods
Least-squares matrix: full	Secondary atom site location: difference Fourier map
*R*[*F*^2^ > 2σ(*F*^2^)] = 0.044	Hydrogen site location: inferred from neighbouring sites
*wR*(*F*^2^) = 0.112	H atoms treated by a mixture of independent and constrained refinement
*S* = 1.09	*w* = 1/[σ^2^(*F*_o_^2^) + (0.0466*P*)^2^ + 3.7642*P*] where *P* = (*F*_o_^2^ + 2*F*_c_^2^)/3
1469 reflections	(Δ/σ)_max_ < 0.001
144 parameters	Δρ_max_ = 1.50 e Å^−^^3^
0 restraints	Δρ_min_ = −0.43 e Å^−^^3^

### Special details

**Table:**

Geometry. All esds (except the esd in the dihedral angle between two l.s. planes) are estimated using the full covariance matrix. The cell esds are taken into account individually in the estimation of esds in distances, angles and torsion angles; correlations between esds in cell parameters are only used when they are defined by crystal symmetry. An approximate (isotropic) treatment of cell esds is used for estimating esds involving l.s. planes.
Refinement. Refinement of *F*^2^ against ALL reflections. The weighted *R*-factor *wR* and goodness of fit *S* are based on *F*^2^, conventional *R*-factors *R* are based on *F*, with *F* set to zero for negative *F*^2^. The threshold expression of *F*^2^ > σ(*F*^2^) is used only for calculating *R*-factors(gt) *etc*. and is not relevant to the choice of reflections for refinement. *R*-factors based on *F*^2^ are statistically about twice as large as those based on *F*, and *R*- factors based on ALL data will be even larger.

### Fractional atomic coordinates and isotropic or equivalent isotropic displacement parameters (Å^2^)

**Table:**

	*x*	*y*	*z*	*U*_iso_*/*U*_eq_	
Co1	0.30993 (8)	−0.38921 (6)	−0.53843 (5)	0.0158 (2)	
O1	0.2961 (5)	−0.4313 (3)	−0.3763 (3)	0.0234 (7)	
N1	0.3036 (6)	−0.1727 (4)	−0.1987 (3)	0.0212 (8)	
N2	0.3051 (6)	−0.3027 (4)	−0.2197 (3)	0.0204 (8)	
N3	0.2971 (5)	−0.3160 (4)	−0.3251 (3)	0.0179 (8)	
N4	0.2849 (6)	−0.1814 (4)	−0.4803 (3)	0.0211 (8)	
N5	0.3650 (6)	−0.5988 (4)	−0.5436 (3)	0.0213 (8)	
N6	0.2624 (6)	−0.6837 (4)	−0.6055 (3)	0.0230 (9)	
N7	0.1639 (8)	−0.7656 (5)	−0.6637 (4)	0.0420 (12)	
C1	0.2719 (7)	−0.0579 (5)	−0.5091 (4)	0.0261 (11)	
H1A	0.2669	−0.0371	−0.5816	0.031*	
C2	0.2650 (9)	0.0474 (5)	−0.4373 (4)	0.0317 (11)	
H2A	0.2529	0.1337	−0.4644	0.038*	
C3	0.2756 (8)	0.0249 (5)	−0.3296 (4)	0.0261 (10)	
H3A	0.2724	0.0936	−0.2817	0.031*	
C4	0.2914 (7)	−0.1065 (5)	−0.2948 (4)	0.0225 (10)	
C5	0.2908 (6)	−0.1985 (4)	−0.3742 (3)	0.0172 (9)	
O1W	−0.0066 (6)	−0.3972 (5)	−0.6162 (4)	0.0402 (10)	
H1WB	−0.045 (10)	−0.364 (7)	−0.673 (6)	0.05 (2)*	
H1WA	−0.078 (12)	−0.466 (8)	−0.626 (7)	0.07 (3)*	

### Atomic displacement parameters (Å^2^)

**Table:**

	*U*^11^	*U*^22^	*U*^33^	*U*^12^	*U*^13^	*U*^23^
Co1	0.0175 (3)	0.0180 (3)	0.0126 (3)	−0.0012 (2)	0.0067 (2)	0.0002 (2)
O1	0.0313 (18)	0.0192 (16)	0.0224 (16)	−0.0006 (14)	0.0135 (14)	−0.0048 (13)
N1	0.0208 (19)	0.027 (2)	0.0158 (18)	0.0013 (16)	0.0071 (15)	0.0004 (16)
N2	0.029 (2)	0.0180 (19)	0.0177 (18)	0.0012 (16)	0.0130 (16)	0.0002 (15)
N3	0.0213 (19)	0.0178 (19)	0.0165 (18)	−0.0005 (15)	0.0094 (15)	−0.0018 (15)
N4	0.0217 (19)	0.029 (2)	0.0139 (18)	0.0027 (16)	0.0087 (15)	0.0016 (16)
N5	0.0205 (19)	0.019 (2)	0.023 (2)	−0.0030 (16)	0.0070 (16)	−0.0041 (16)
N6	0.026 (2)	0.023 (2)	0.0179 (19)	−0.0030 (18)	0.0063 (17)	0.0031 (18)
N7	0.050 (3)	0.035 (3)	0.031 (2)	−0.018 (2)	0.004 (2)	−0.006 (2)
C1	0.032 (3)	0.033 (3)	0.020 (2)	−0.006 (2)	0.017 (2)	−0.004 (2)
C2	0.049 (3)	0.023 (3)	0.026 (3)	−0.001 (2)	0.017 (2)	0.004 (2)
C3	0.037 (3)	0.022 (2)	0.022 (2)	0.000 (2)	0.014 (2)	−0.0031 (19)
C4	0.022 (2)	0.027 (3)	0.019 (2)	0.0011 (19)	0.0094 (18)	0.0003 (19)
C5	0.018 (2)	0.020 (2)	0.014 (2)	−0.0006 (17)	0.0074 (17)	−0.0007 (17)
O1W	0.0229 (19)	0.051 (3)	0.037 (2)	−0.0086 (18)	0.0013 (17)	0.021 (2)

### Geometric parameters (Å, °)

**Table:**

Co1—O1W	2.069 (4)	N4—C5	1.341 (5)
Co1—N1^i^	2.111 (4)	N5—N6	1.198 (5)
Co1—N5^ii^	2.128 (4)	N5—Co1^ii^	2.128 (4)
Co1—O1	2.140 (3)	N6—N7	1.147 (6)
Co1—N5	2.163 (4)	C1—C2	1.416 (7)
Co1—N4	2.259 (4)	C1—H1A	0.9300
O1—N3	1.334 (5)	C2—C3	1.357 (7)
N1—N2	1.343 (5)	C2—H2A	0.9300
N1—C4	1.365 (6)	C3—C4	1.392 (7)
N1—Co1^iii^	2.111 (4)	C3—H3A	0.9300
N2—N3	1.323 (5)	C4—C5	1.371 (6)
N3—C5	1.335 (6)	O1W—H1WB	0.74 (7)
N4—C1	1.295 (6)	O1W—H1WA	0.84 (8)
			
O1W—Co1—N1^i^	86.72 (16)	C1—N4—Co1	144.3 (3)
O1W—Co1—N5^ii^	174.45 (17)	C5—N4—Co1	103.4 (3)
N1^i^—Co1—N5^ii^	95.61 (15)	N6—N5—Co1^ii^	122.8 (3)
O1W—Co1—O1	90.03 (15)	N6—N5—Co1	130.7 (3)
N1^i^—Co1—O1	173.20 (13)	Co1^ii^—N5—Co1	102.40 (15)
N5^ii^—Co1—O1	88.16 (14)	N7—N6—N5	179.2 (5)
O1W—Co1—N5	97.02 (17)	N4—C1—C2	124.2 (4)
N1^i^—Co1—N5	101.43 (15)	N4—C1—H1A	117.9
N5^ii^—Co1—N5	77.60 (15)	C2—C1—H1A	117.9
O1—Co1—N5	84.88 (13)	C3—C2—C1	121.3 (5)
O1W—Co1—N4	89.00 (17)	C3—C2—H2A	119.4
N1^i^—Co1—N4	93.61 (14)	C1—C2—H2A	119.4
N5^ii^—Co1—N4	95.87 (14)	C2—C3—C4	116.4 (4)
O1—Co1—N4	80.35 (12)	C2—C3—H3A	121.8
N5—Co1—N4	164.06 (14)	C4—C3—H3A	121.8
N3—O1—Co1	107.4 (2)	N1—C4—C5	107.7 (4)
N2—N1—C4	107.9 (4)	N1—C4—C3	136.2 (4)
N2—N1—Co1^iii^	118.9 (3)	C5—C4—C3	116.1 (4)
C4—N1—Co1^iii^	133.2 (3)	N3—C5—N4	124.4 (4)
N3—N2—N1	107.4 (3)	N3—C5—C4	105.8 (4)
N2—N3—O1	124.8 (3)	N4—C5—C4	129.8 (4)
N2—N3—C5	111.2 (3)	Co1—O1W—H1WB	111 (5)
O1—N3—C5	124.0 (3)	Co1—O1W—H1WA	125 (5)
C1—N4—C5	112.2 (4)	H1WB—O1W—H1WA	105 (7)
			
O1W—Co1—O1—N3	−93.8 (3)	N5^ii^—Co1—N5—Co1^ii^	0.0
N1^i^—Co1—O1—N3	−32.4 (12)	O1—Co1—N5—Co1^ii^	−89.23 (15)
N5^ii^—Co1—O1—N3	91.4 (3)	N4—Co1—N5—Co1^ii^	−67.1 (5)
N5—Co1—O1—N3	169.1 (3)	Co1^ii^—N5—N6—N7	91 (38)
N4—Co1—O1—N3	−4.9 (2)	Co1—N5—N6—N7	−116 (38)
C4—N1—N2—N3	0.9 (5)	C5—N4—C1—C2	0.0 (7)
Co1^iii^—N1—N2—N3	179.0 (3)	Co1—N4—C1—C2	−177.8 (4)
N1—N2—N3—O1	179.9 (4)	N4—C1—C2—C3	1.3 (8)
N1—N2—N3—C5	0.1 (5)	C1—C2—C3—C4	−0.6 (8)
Co1—O1—N3—N2	−174.8 (3)	N2—N1—C4—C5	−1.5 (5)
Co1—O1—N3—C5	4.9 (5)	Co1^iii^—N1—C4—C5	−179.3 (3)
O1W—Co1—N4—C1	−87.4 (6)	N2—N1—C4—C3	176.7 (5)
N1^i^—Co1—N4—C1	−0.8 (6)	Co1^iii^—N1—C4—C3	−1.1 (8)
N5^ii^—Co1—N4—C1	95.3 (6)	C2—C3—C4—N1	−179.2 (5)
O1—Co1—N4—C1	−177.6 (6)	C2—C3—C4—C5	−1.1 (7)
N5—Co1—N4—C1	160.0 (5)	N2—N3—C5—N4	179.1 (4)
O1W—Co1—N4—C5	94.7 (3)	O1—N3—C5—N4	−0.7 (6)
N1^i^—Co1—N4—C5	−178.7 (3)	N2—N3—C5—C4	−1.1 (5)
N5^ii^—Co1—N4—C5	−82.7 (3)	O1—N3—C5—C4	179.1 (4)
O1—Co1—N4—C5	4.5 (3)	C1—N4—C5—N3	177.6 (4)
N5—Co1—N4—C5	−17.9 (6)	Co1—N4—C5—N3	−3.7 (5)
O1W—Co1—N5—N6	24.1 (4)	C1—N4—C5—C4	−2.2 (7)
N1^i^—Co1—N5—N6	−64.0 (4)	Co1—N4—C5—C4	176.5 (4)
N5^ii^—Co1—N5—N6	−157.3 (5)	N1—C4—C5—N3	1.6 (5)
O1—Co1—N5—N6	113.5 (4)	C3—C4—C5—N3	−177.0 (4)
N4—Co1—N5—N6	135.6 (5)	N1—C4—C5—N4	−178.6 (4)
O1W—Co1—N5—Co1^ii^	−178.63 (17)	C3—C4—C5—N4	2.8 (7)
N1^i^—Co1—N5—Co1^ii^	93.31 (16)		

Symmetry codes: (i) *x*, −*y*−1/2, *z*−1/2; (ii) −*x*+1, −*y*−1, −*z*−1; (iii) *x*, −*y*−1/2, *z*+1/2.

### Hydrogen-bond geometry (Å, °)

**Table:**

*D*—H···*A*	*D*—H	H···*A*	*D*···*A*	*D*—H···*A*
O1W—H1WB···N7^iv^	0.74 (7)	2.15 (7)	2.894 (6)	178 (7)
O1W—H1WA···O1^v^	0.84 (8)	1.87 (8)	2.661 (5)	156 (8)

Symmetry codes: (iv) −*x*, *y*+1/2, −*z*−3/2; (v) −*x*, −*y*−1, −*z*−1.
